# PI3K Contributed to Modulation of Spinal Nociceptive Information Related to ephrinBs/EphBs

**DOI:** 10.1371/journal.pone.0040930

**Published:** 2012-08-03

**Authors:** Li-Na Yu, Xue-Long Zhou, Jing Yu, Hao Huang, Li-Shan Jiang, Feng-Jiang Zhang, Jun-Li Cao, Min Yan

**Affiliations:** 1 Department of Anesthesiology, The Second Affiliated Hospital, School of Medicine, Zhejiang University, Hangzhou, Zhejiang, People's Republic of China; 2 Jiangsu Province Key Laboratory of Anesthesiology and Center for Pain Research and Treatment, Xuzhou Medical College, Xuzhou, Jiangsu, People's Republic of China; University of Cincinnatti, United States of America

## Abstract

There is accumulating evidence to implicate the importance of EphBs receptors and ephrinBs ligands were involved in modulation of spinal nociceptive information. However, the downstream mechanisms that control this process are not well understood. In the present study, we investigated whether phosphatidylinositol 3-kinase (PI3K), as the downstream effectors, participates in modulation of spinal nociceptive information related to ephrinBs/EphBs. Intrathecal injection of ephrinB1-Fc produced a dose- and time-dependent thermal and mechanical hyperalgesia, accompanied by the increase of spinal PI3K-p110γ, phosphorylation of AKT (p-AKT) and c-Fos expression. Pre-treatment with PI3K inhibitor wortmannin or LY294002 prevented activation of spinal AKT induced by ephrinB1-Fc. Inhibition of spinal PI3K signaling dose-dependently prevented and reversed pain behaviors and spinal c-Fos protein expression induced by intrathecal injection of ephrinB1-Fc. Inhibition of EphBs receptors by intrathecal injection of EphB1-Fc reduced formalin-induced inflammation and chronic constrictive injury-induced neuropathic pain behaviors accompanied by decreased expression of spinal PI3K,p-AKT and c-Fos protein. Furthermore, pre-treatment with PI3K inhibitor wortmannin or LY294002 prevented ephrinB1-Fc-induced ERK activation in spinal. These data demonstrated that PI3K and PI3K crosstalk to ERK signaling contributed to modulation of spinal nociceptive information related to ephrinBs/EphBs.

## Introduction

Central sensitization, an activity-dependent functional plasticity in spinal cord neurons, is one of the main causes of behavior hyperalgesia under pathologic conditions and has been under intensive investigation [1], [2], [3]. Activation of postsynaptic membrane receptors or ion channels, intracellular kinase cascades, and intranuclear gene expression contributes to the induction, development, and maintenance of central sensitization. Eph receptor tyrosine kinases and their membranebound ligands, ephrins, which are the largest family of receptor tyrosine kinase(RTK)system, are involved in diverse aspects of development, such as tissue patterning, angiogenesis, axon guidance, and synapse formation [4], [5], [6], [7]. Recent advances indicate that Eph receptors and ephrin ligands are present in the adult brain and peripheral tissue and play a critical role in modulating multiple aspects of physiology and pathophysiology(e.g., activity-dependent synaptic plasticity, regulation of pain threshold, epileptogenesis, inflammation response, and excitotoxic neuronal death) [8], [9], [10], [11]. Interestingly, several Eph receptors and ephrin ligands are also expressed in the adult rat spinal cord and the dorsal root ganglion [12], [13], [14]. Bundesen et al. [12] reported that EphB2 receptor was present in the laminae I–III of the dorsal horn and on small- and medium-sized dorsal root ganglion neurons but not on large-diameter neurons, two important sites for modulation of nociceptive information. Recently, some studies also demonstrated that activation of spinal ephrinBs/EphBs system played a critical role in the development and maintenance of chronic pain after peripheral nerve injury [15], [16], [17].These studies indicated that Ephrin/Eph system may be involved in physiologic and pathologic pain modulation in the spinal cord level. However, the downstream mechanisms that control this process are not well understood.

PI3K, which phosphorylates the D3 position of the inositol ring of phosphoinositides and thereby generates intracellular signaling molecules, has been demonstrated to be essential for a plethora of physiological and pathological processes [18], [19], [20]. It is well established that activation of PI3K signaling is involved in the modulation of nociceptive information and central sensitization produced by intense noxious stimuli [21], [22], [23], [24], [25], [26], . Importantly, PI3K is proposed to mediate central sensitization and hyperalgesia induced by activation of central RTK system NGF/TrkA, BDNF/TrkB and G-CSF/G-CSFR signaling [22], [23], [25]. Moreover, several lines of evidence have shown that regulation of PI3K pathway is associated with EphBs receptors activation in other study fields, such as intraplantar injection of ephrinB1-Fc induced pain [28]and retinal endothelial cell [29] and microvascular endothelial cell migration and proliferation [30]. In the present study, we hypothesized that like other RTK systems, pain behaviors induced by activation of spinal ephrinBs/EphBs signaling were mediated by activation of spinal PI3K and we provide strong evidence to support this hypothesis.

## Results

### Intrathecal injection of ephrinB1-Fc induced a time- and dose-dependent hyperalgesia and spinal Fos expression

**Figure 1:**
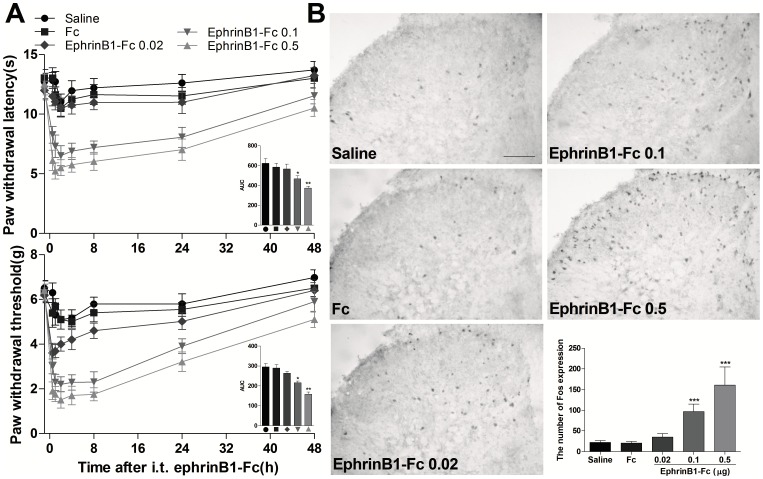
Intrathecal (i.t.) injection of ephrinB1-Fc produced a dose- and time-dependent hyperalgesia and spinal Fos protein expression. Paw withdrawal latency to the radiant heat and paw withdrawal threshold to mechanical stimuli were recorded at 1-h, 2-h, 4-h, 8-h, 24-h, and 48-h after ephrinB1-Fc injection. Spinal Fos protein expression was assayed at 2-h after ephrinB1-Fc injection. (A)Intrathecal injection of 0.1 or 0.5 µg ephrinB1-Fc, not 0.02 µg ephrinB1-Fc or 0.5 µg control Fc, produced thermal hyperalgesia and mechanical allodynia. *P*<0.05, from 0.5 to 24 h time point, ephrinB1-Fc 0.1 or ephrinB1-Fc 0.5 group compared with saline or Fc group. Inset figures showed that the calculated area under the curve (AUC) (−2–48 h) in paw-withdrawal latency (PWL) and paw-withdrawal threshold (PWT) test was significantly decreased in a dose-dependent manner in ephrinB1-Fc group. *P<0.05, **P<0.01 compared with saline or Fc group. n = 8 mice in each group. (B) Representative immunohistochemical staining and the quantitative data of Fos expression in the spinal cord of mice. Intrathecal injection of ephrinB1-Fc (0.02, 0.1, and 0.5 µg), not saline or Fc (0.5 µg), induced a dose-dependent increase in spinal Fos protein expression in superficial lamine of ipsilateral spinal cord,*** *P*<0.001 compared with saline group, n = 6 mice in each group. Scale bar = 100 µm.

In the present study, we found that intrathecal injection of ephrinB1-Fc (0.02, 0.1, and 0.5 µg in 5 µl saline), not control Fc, induced a dose-dependent thermal hyperalgesia and mechanical allodynia in mice, which can last at least up to 24 h and return to baseline level on 48 h after injection of ephrinB1-Fc (*P*<0.05, from 0.5 to 24 h time point, ephrinB1-Fc 0.1 or ephrinB1-Fc 0.5 group compared with saline or Fc group; fig. 1A). Compared with saline and Fc groups, the calculated area under the curve (−2–48 h) in PWL and PWT tests was significantly decreased in a dose-dependent manner in ephrinB1-Fc group (fig. 1A inset).To rule out a nonspecific effect through the Fc portion, human Fc was used as a control. No significant hyperalgesia was detected after injection of human Fc. these results demonstrated that activation of spinal EphBs receptors could produce thermal hyperalgesia, mechanical allodynia. Although, so far, we still cannot identify which EphBs subgroup was activated dominantly by ephrinB1-Fc.

Spinal neuronal sensitization was involved in the development and maintenance of hyperalgesia induced by the different causes. Fos protein, the product of c-fos immediate early gene, has been used as a maker for neuronal activation in the central nervous system [31], [32]. There is a positive correlation between the quantity of Fos protein expression and the neuronal activation induced by nociceptive stimuli in spinal cord neurons. To further clarify the algesic effect of intrathecal injection of ephrinB1-Fc, we investigated the change of spinal Fos protein expression following intrathecal injection of different doses of ephrinB1-Fc (0.02, 0.1, and 0.5 µg in 5 µl saline). Intrathecal injection of ephrinB1-Fc, not saline or Fc, induced a dose-dependent increase in spinal Fos protein expression. The expression of Fos protein mainly distributed in I–V lamina of spinal cord (*P*<0.001, ephrinB1-Fc 0.1 or ephrinB1-Fc 0.5 group compared with saline or Fc group; fig. 1B). These results further confirmed that activation of spinal ephrinBs/EphBs system can sensitize the spinal neurons and induce pain behaviors in naïve mice.

### Intrathecal injection of ephrinB1-Fc induced a time-dependent and dose-dependent activation of spinal PI3K and AKT

**Figure 2:**
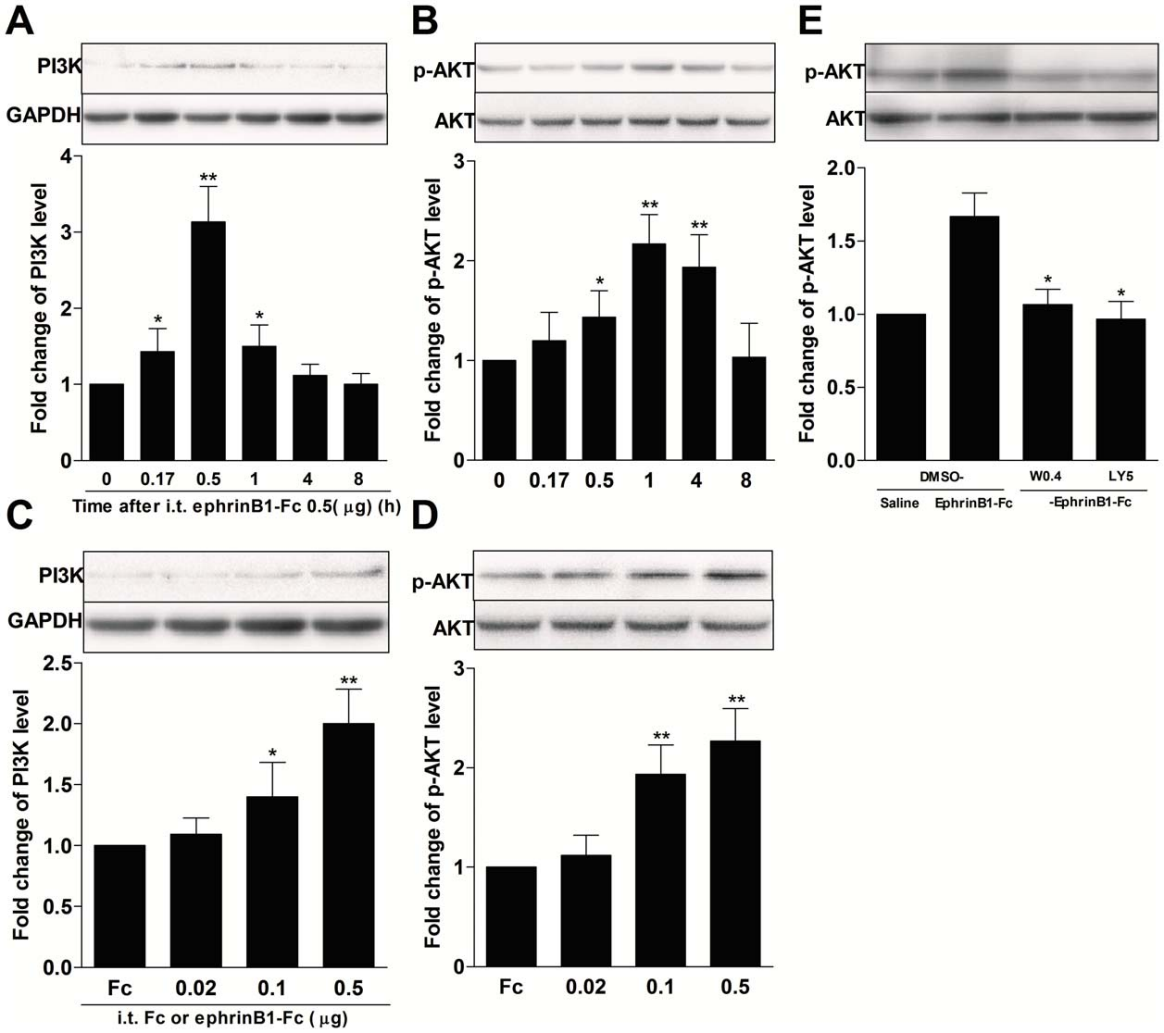
Intrathecal(i.t.) injection of ephrinB1-Fc induced a dose- and time-dependent increase of spinal PI3K-p110γ and p-AKT expression. The expression of spinal PI3K-p110γ and p-AKT was assayed at 0, 0.17-h, 0.5-h, 1-h, 4-h, and 8-h time-points in the time-dependent experiments and at 0.5-h time-point in the dose-dependent experiments after ephrinB1-Fc injection. PI3K inhibitor wormannin (0.4 µg) or LY294002 (5 µg) or DMSO was intrathecally injected at 30 min before i.t. ephrinB1-Fc (0.5 µg),the expression of p-AKT in spinal was assayed at 1 h after ephrinB1-Fc injection in PI3K mediated spinal AKT activation experiment. (A, B) The representative bands (top) for the expression of PI3K-p110γ or p-AKT in spinal at different time points after injection of ephrinB1-Fc (0.5 µg) and the quantitative data (bottom) for the expression of PI3K-p110γ or p-AKT. The fold change for the density of PI3K-p110γ or p-AKT normalized to GAPDH or AKT for each sample, respectively. The fold change of PI3K-p110γ or p-AKT level in 0 time point groups was set at 1 for quantifications. *P<0.05, **P<0.01, compared with 0 time point group, n = 4 mice in each group. (C, D) The representative bands (top) for the expression of PI3K-p110γ or p-AKT in spinal after injection of various dose of ephrinB1-Fc (0.02, 0.1, and 0.5 µg). The quantitative data (bottom) for the expression of PI3K-p110γ or p-AKT. The fold change for the density of PI3K-p110γ or p-AKT normalized to GAPDH or AKT for each sample, respectively. The fold change of PI3K-p110γ or p-AKT level in Fc groups was set at 1 for quantifications. *P<0.05, **P<0.01, compared with Fc group, n = 4 mice in each group.(E) The representative bands (top) and the quantitative data (bottom) for the expression of spinal p-AKT. The fold change for the density of p-AKT normalized to AKT for each sample. The fold change of p-AKT level in DMSO-saline group was set at 1 for quantifications.*P<0.05, compared with DMSO-ephrinB1-Fc group, n = 4 mice in each group.

Some studies have shown that spinal PI3K activation mediated inflammatory or injury-induced spinal sensitization and hyperalgesia [21], [22], [23], [24], [25], [26], [27]. Furthermore, PI3K also mediated central sensitization and hyperalgesia induced by activation of central RTK system NGF/TrkA and G-CSF/G-CSFR signaling [22], [23], [25]. Thus, we want to know if ephrinBs/EphBs signaling, like other RTK pathways such as NGF/TrkA and C-GSF/C-GSFR, is involved in the regulation of the central sensitization through a PI3K-dependent mechanism. To determine whether intrathecal(i.t.) injection of ephrinB1-Fc activates PI3K/AKT pathway, we measured the time course and dose-dependent relationship of expression of PI3K and phosphorylation of downstream kinase AKT (S473) as an indicator of activation of PI3K pathway in the spinal. As shown in fig. 2, i.t. injection of ephrinB1-Fc (0.5 µg) caused an increase of PI3K-p110γ on already detectable 10 min after injection and reaching maximal level on 30 min and returning to baseline level on 8 h after injection (PI3K-p110γ: *P*<0.05 at 0.17 h, *P*<0.01 at 0.5 h, compared with 0 h group; fig. 2A). The same injection increase the expression of phosphorylation of AKT on already detectable 30 min after injection, which reached its peak by 1 h and lasted at least up to 8 h after injection (p-AKT: *P*<0.05 at 0.5 h, *P*<0.01 at 1 h, compared with 0 h group; fig. 2B). To further determine ephrinB1-induced activation of PI3K/AKT pathway, we picked up 30-min time point to perform dose–response experiments. I.t. injection of ephrinB1-Fc (0.02, 0.1, and 0.5 µg) produced a dose-dependent increase of spinal PI3K-p110γ expression and of phosphorylation of AKT (*P*<0.05 or *P*<0.01, ephrinB1-Fc 0.1 or ephrinB1-Fc 0.5 group compared with Fc group; fig. 2C, 2D). To explore the role of PI3K in AKT activation by ephrinB1-Fc, we intrathecally injected wortmannin, an irreversible PI3K inhibitor, or LY294002, a reversible competitive PI3K inhibitor, at 30 min before ephrinB1-Fc injection, then tested the expression of spinal p-AKT at 1 h after ephrinB1-Fc injection. We found that both pretreatments prevented the increase of p-AKT expression (*P<0.05*, compared with DMSO-ephrinB1-Fc group; fig. 2E). It suggested that PI3K contributed to the activation of spinal AKT by ephrinB1-Fc. In our previous experiments, to exclude a nonspecial effect of the Fc portion, human Fc was used as a control for ephrinB1-Fc. We found that no significant hyperalgesia was detected after injection of human Fc. Therefore, vehicle for ephrinB1-Fc was used as its control in the present study.

### Inhibition of spinal PI3K prevented and reversed pain behaviors induced by intrathecal injection of ephrinB1-Fc

**Figure 3:**
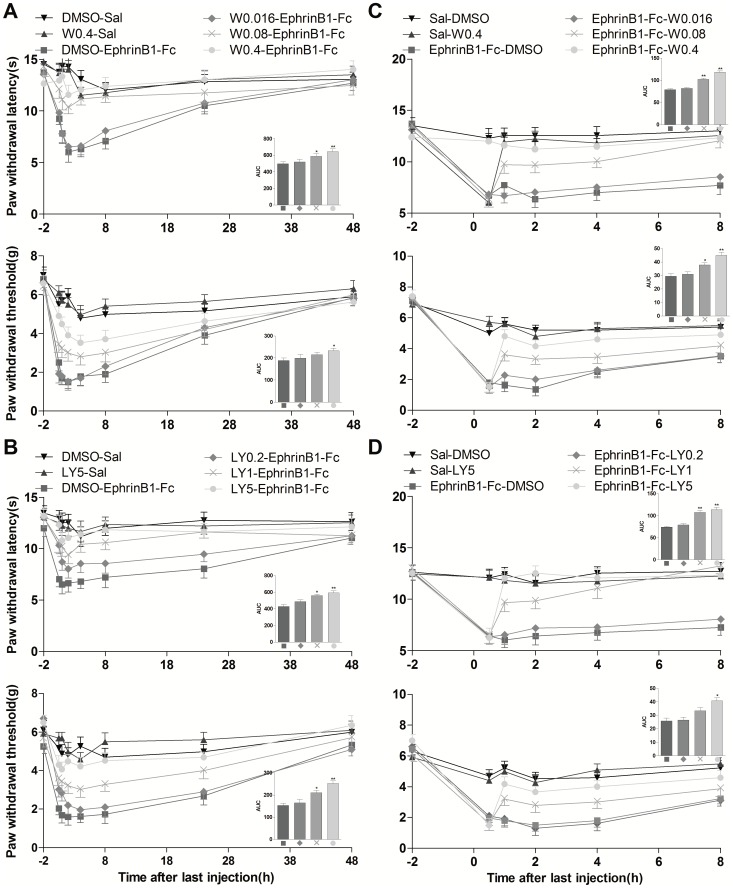
Inhibition of PI3K prevented and reversed pain behavior induced by i.t. ephrinB1-Fc. PI3K inhibitors were injected at 0.5-h before or after ephrinB1-Fc injection. Paw withdrawal latency to the radiant heat and paw withdrawal threshold to mechanical stimuli were recorded at 0.5-h, 1-h, 2-h, 4-h, 8-h, 24-h and 48-h after ephrinB1-Fc (0.5 µg) injection in the pre-treatment experiment and at 0.5-h, 1-h, 2-h, 4-h, and 8-h after PI3K inhibitors in post-treatment experiment. (A,B) Pre-treatment with various dose of wortmannin (0.016, 0.08, and 0.4 µg) or LY294002 (0.2, 1, and 5 µg) prevented ephrinB1-Fc-induced thermal hyperalgesia (Top) and mechanical allodynia (bottom) in a dose-dependent manner. The calculated area under the curve (AUC) (−2–24 h) (inside bar figure) was significantly increased in W0.4-, W0.08-, LY5- and LY1-ephrinB1-Fc group in PWL test, and in W0.4-, LY5- or LY1-ephrinB1-Fc group in PWT test, *P<0.05, **P<0.01 when compared with DMSO-ephrinB1-Fc group, n = 8 mice in each group. (C,D) Post-treatment with wortmannin (0.016, 0.08, and 0.4 µg) or LY294002 (0.2, 1, and 5 µg) dose-dependently reversed the established thermal hyperalgesia (top) and mechanical allodynia (bottom) by ephrinB1-Fc (0.5 µg).Compared with ephrinB1-Fc-DMSO group, the calculated area under the curve (AUC) (−2–8 h) (inside bar figure) was significantly increased in ephrinB1-Fc-W0.4-, -W0.08, –LY5,or –LY1 group in PWL test, and in ephrinB1-Fc-W0.4, -W0.08,or –LY5 group in PWT test, *P<0.05, **P<0.01 compared with ephrinB1-Fc-DMSO group, n = 8 mice in each group.

The current data have indicated that intrathecal injection of ephrinB1-Fc induced thermal hyperalgesia and mechanical allodynia, which was associated with activation of spinal PI3K pathway. Therefore, inhibition of PI3K should alleviate ephrinB1-Fc induced pain behaviors in theory. As we expected, pre-treatment with wortmannin (0.016, 0.08, and 0.4 µg in 1% DMSO) or LY294002 (0.2, 1, and 5 µg in 1% DMSO), not DMSO, at 0.5 h before i.t. injection of ephrinB1-Fc (0.5 µg),dose-dependently prevented thermal hyperalgesia and mechanical allodynia induced by i.t. injection of ephrinB1-Fc (0.5 µg). Compared with DMSO-ephrinB1-Fc group, PWL and PWT were significantly elevated in W0.4-ephrinB1-Fc and LY5-ephrinB1-Fc group at 0.5–8 h after i.t. injection of ephrinB1-Fc (*P*<0.05 or *P*<0.01) (fig. 3A, 3B). The calculated area under curve (AUC) (−2–48 h)was significantly increased in W0.4-,W0.08-,LY5- or LY1-ephrinB1-Fc group in PWL test, and in W0.4-,LY5- or LY1-ephrinB1-Fc group in PWT test (*P*<0.05 or *P*<0.01) (fig. 3A, 3B inset). These results suggested that activation of PI3K pathway was involved in the initiation of pain behaviors induced by i.t. injection of ephrinB1-Fc. Then we asked if PI3K pathway also participated in its maintenance process. To address this question, wortmannin (0.016, 0.08, and 0.4 µg) or LY294002 (0.2, 1,and 5 µg) was administrated at 0.5 h after i.t. injection of ephrinB1-Fc(0.5 µg).We found that post-treatment with both PI3K inhibitors dose-dependently reversed the established thermal hyperalgesia and mechanical allodynia by i.t. injection of ephrinB1-Fc (*P*<0.05 or *P*<0.01) (fig. 3C, 3D).Compared with ephrinB1-Fc-DMSO group, the calculated area under the curve (AUC) (−2–8 h) (inside bar figure)was significantly increased in ephrinB1-Fc-W0.4, -W0.08,–LY5 or LY1 group in PWL test and in ephrinB1-Fc-W0.4, -W0.08,or –LY5 in PWT test (*P*<0.05 or *P*<0.01) (fig. 3C, 3D inset), suggesting that PI3K pathway is required for the maintenance of pain behaviors induced by i.t. injection of ephrinB1-Fc. This effect lasted at least up to 8 h after injection of PI3K inhibitors.

### Inhibition of spinal PI3K prevented and reversed spinal Fos protein expression induced by intrathecal injection of ephrinB1-Fc

**Figure 4:**
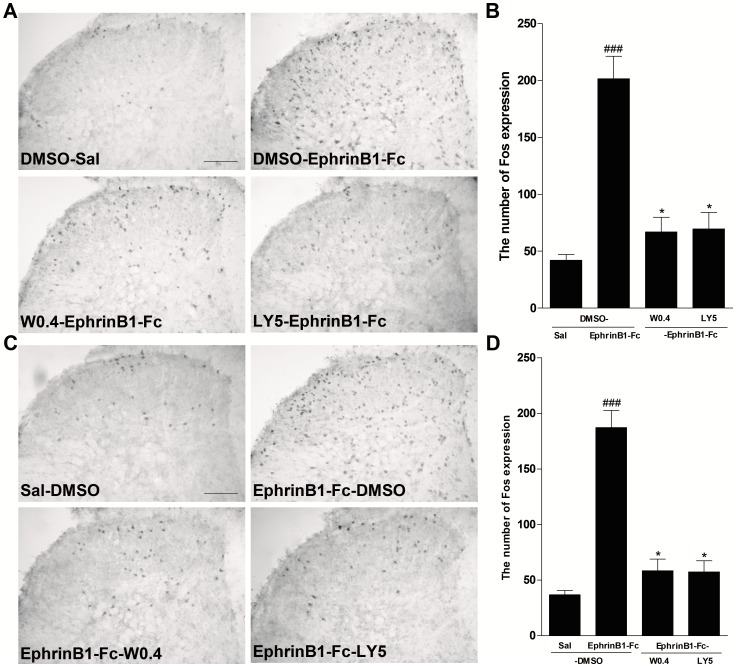
Inhibition of PI3K partially inhibited or reversed the increased expression of spinal Fos protein by i.t. ephrinB1-Fc. PI3K inhibitor wortmannin (0.4 µg) or LY294002 (5 µg) or DMSO was intrathecally injected at 30 min before and after i.t. ephrinB1-Fc (0.5 µg). Spinal Fos protein expression was assayed at 2 h after ephrinB1-Fc injection. (A,C) Representative Fos immunohistochemical staining and (B,D) the quantitative data of Fos expression in the spinal cord of mice in pre-treatment experiments or post-treatment experiments. ###*P*<0.001, compared with Sal-DMSO or DMSO-Sal group, *P<0.05, compared with DMSO-ephrinB1-Fc or ephrinB1-Fc-DMSO group, n = 6 mice in each group, Scale bar = 100 µm.

The expression of Fos protein also may be a useful tool to examine the effectiveness of different analgesic regimens [31], [32]. To further clarify the analgesic effect of inhibition of PI3K on pain induced by i.t. injection of ephrinB1-Fc, we investigated the effect of pre-treatment or post-treatment with PI3K inhibitors on spinal Fos protein expression induced by i.t. injection of ephrinB1-Fc (0.5 µg). DMSO, wortmannin (0.4 µg) or LY294002 (5 µg) was intrathecally administrated at 30 min before or after i.t. injection of ephrinB1-Fc (0.5 µg) and spinal Fos protein expression was assayed at 2 h after ephrinB1-Fc injection. The results showed that both pre-treatment and post-treatment with both PI3K inhibitors partially inhibited the spinal Fos expression induced by i.t. injection of ephrinB1-Fc (*P*<0.05, compared with DMSO-ephrinB1-Fc or ephrinB1-Fc-DMSO group; fig. 4).

### EphBs receptors were involved in activation of spinal PI3K and AKT in the inflammatory pain model

**Figure 5:**
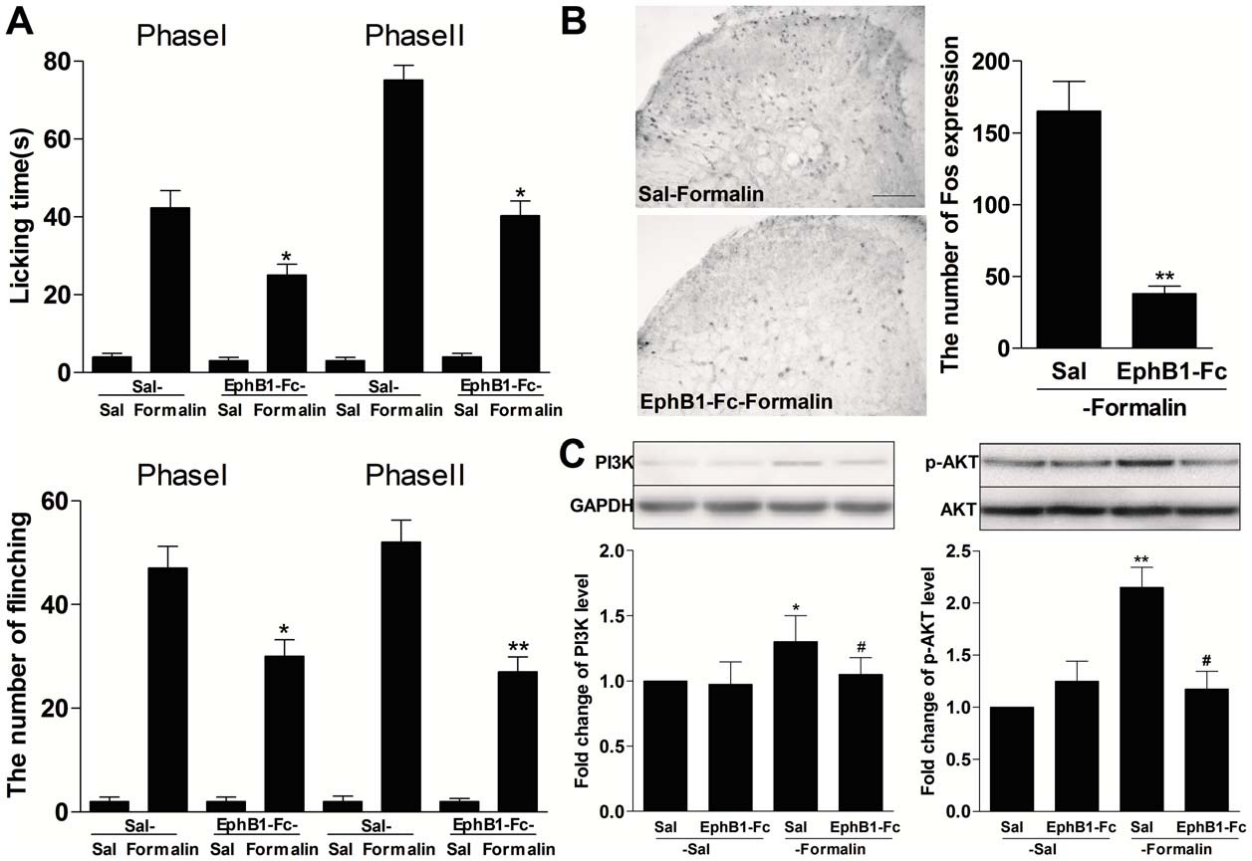
Intrathecal (i.t.) pretreatment with EphB1-Fc inhibited formalin-induced inflammatory pain and spinal Fos protein expression, accompanied by the decreased expression of spinal PI3K-p110γ and phosphorylated AKT (p-AKT). The amount of time spent licking the injected paw and the number of flinching paw were measured from 0- to 5-min (the first phase) and from 10- to 40-min (the second phase) after formalin injection. EphB1-Fc was injected at 30-min before formalin injection. The expression of spinal Fos protein and spinal PI3K-p110γ and p-AKT was measured at 2-h and 30-min after injection of formalin, respectively. (A) Pretreatment of EphBs receptors blocker EphB1-Fc (0.5 µg) decreased the time of licking paw and the number of flinching paw induced by formalin. *P<0.05 or **P<0.01 compared with saline (Sal)-Formalin group, n = 8 mice in each group. (C) The representative immunohistochemical staining and the quantitative data for the decreased formalin-induced Fos expression by pretreatment with EphB1-Fc (0.5 µg), not saline, in the spinal cord of mice. ** *P*<0.01 compared with Sal-Formalin group, n = 6 mice in each group, Scale bar = 100 µm. (D) The representative bands and the quantitative data for the decreased expression of spinal PI3K-p110γ and p-AKT by injection of formalin after pretreatment with EphB1-Fc (0.5 µg). The fold change for the density of PI3K-p110γ and p-AKT normalized to GAPDH or AKT for each sample. The fold change of PI3K-p110γ and p-AKT levels in Sal-Sal group was set at 1 for quantifications. *P<0.05 compared with Sal-Sal group, ^#^ *P*<0.05 compared with Sal-Formalin group n = 4 mice in each group.

Spinal ephrinBs or EphBs signaling played a critical role in the development and maintenance of pathological pain. To test whether spinal PI3K and AKT activation contribute to the role of ephrinBs or EphBs signaling in inflammatory pain, we analyzed the effect of intrathecal injection of EphB1-Fc, which can block ephrinBs or EphBs pathway, on expression of spinal PI3K-p110γ and p-AKT in inflammatory models (fig. 5). Pretreatment with intrathecal injection of EphB1-Fc (0.5 µg/5 µl) at 30 min before formalin injection significantly reduced the time of licking and the number of flinching hind paw in phase I and II responses (EphB1-Fc-Formalin group compared with Sal-Formalin group, *P*<0.05 at phase I and II; fig. 5A). The spinal Fos protein (*P*<0.01 compared with Sal-Formalin group; fig. 5B), PI3K-p110γ and p-AKT (PI3K-p110γ: Sal-Formalin group compared with Sal-Sal group: *P*<0.05; EphB1-Fc-Formalin group compared with Sal-Formalin group: *P*<0.05;p-AKT: Sal-Formalin group compared with Sal-Sal group: *P*<0.01; EphB1-Fc-Formalin group compared with Sal-Formalin group: *P*<0.05; fig. 5C) expression, detected at 2 h and 30 min after injection of formalin, respectively, also was markedly inhibited.

### EphBs receptors were involved in activation of spinal PI3K and AKT in the neuropathic pain model

**Figure 6:**
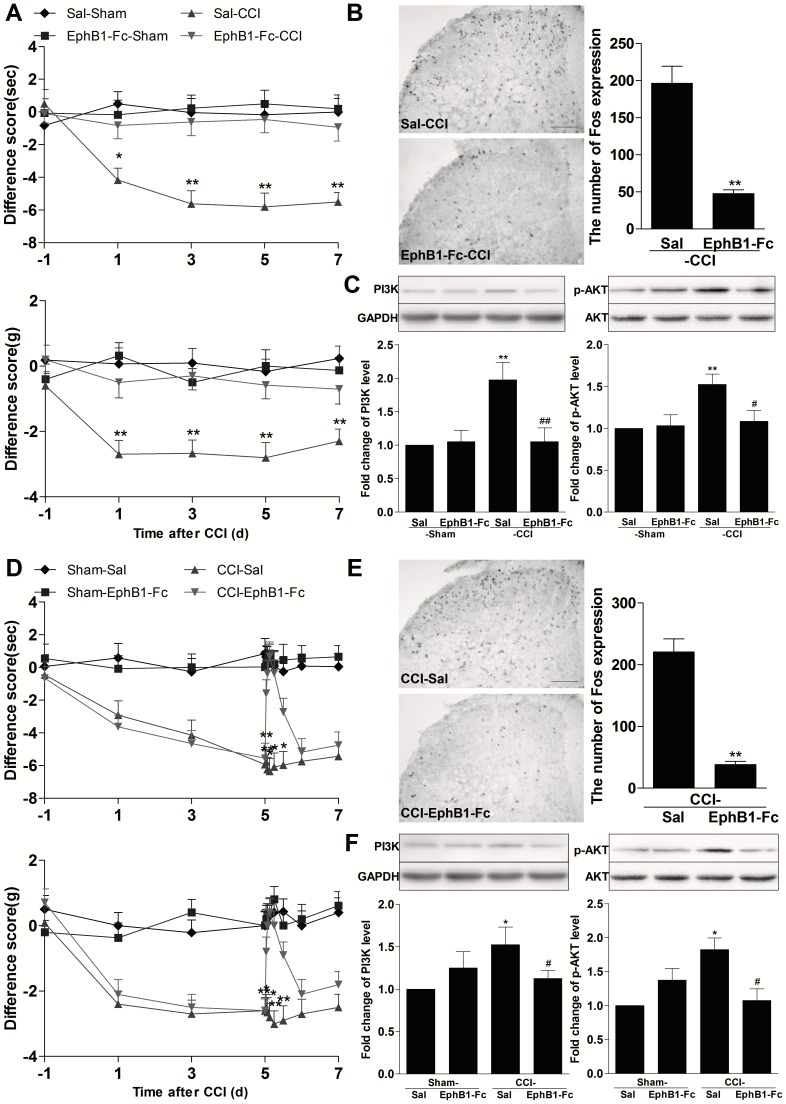
Intrathecal (i.t.) pre- or posttreatment with EphB1-Fc inhibited chronic constrictive injury (CCI)-induced neuropathic pain and spinal Fos protein expression, accompanied by the decreased expression of spinal PI3K-p110γ and phosphorylated AKT (p-AKT). Consecutive intrathecal injection of EphB1-Fc on 1-h before CCI and 1-d and 2-d after CCI in the pre-treatment experiment and Intrathecal injection of a single dose of EphB1-Fc on 5-d after CCI in post-treatment experiment were performed. Paw withdrawal latency to the radiant heat and paw withdrawal threshold to mechanical stimuli were recorded on 1-d, 3-d, 5-d, 7-d after CCI in pre-treatment experiment and on 1-d, 3-d, 0.5-h,1-h,2-h,4-h,8-h after EphB1-Fc injection on 5-d,6-d and 7-d after CCI in post-treatment experiment. The expression of spinal Fos, PI3K-p110γ and p-AKT was assayed on 5-d after CCI in pre-treatment experiment and at 2-h after EphB1-Fc injection on 5-d after CCI in post-treatment experiment.(A) Pretreatment of EphB1-Fc (0.5 µg) prevented CCI-induced thermal hyperalgesia in paw-withdrawal latency (PWL) test and mechanical allodynia in paw-withdrawal threshold (PWT)test. * *P*<0.05, ** *P*<0.01, compared with EphB1-Fc-CCI, n = 8 mice in each group. (B) The representative immunohistochemical staining and the quantitative data for the decreased CCI-induced spinal Fos expression by pretreatment with EphB1-Fc. ** *P*<0.01 compared with saline (Sal)-CCI group, n = 6 mice in each group, Scale bar = 100 µm. (C) The representative bands and the quantitative data for the decreased expression of spinal PI3K-p110γ and p-AKT by CCI after pretreatment with EphB1-Fc. The fold change for the density of PI3K-p110γ and p-AKT normalized to GAPDH or AKT for each sample. The fold change of PI3K-p110γ and p-AKT levels in Sal-Sham group were set at 1 for quantifications. * *P*<0.05 or ** *P*<0.01 compared with Sal -Sham group, ^#^ *P*<0.05 compared with Sal-CCI group, n = 4 mice in each group. (D)Posttreatment of EphB1-Fc reversed CCI-induced thermal hyperalgesia and mechanical allodynia. * *P*<0.05, compared with CCI-EphB1-Fc, n = 8 mice in each group. (E) The reversed CCI-induced spinal Fos expression by posttreatment with EphB1-Fc. ** *P*<0.01 compared with CCI-Sal group, n = 6 mice in each group. (F) The decreased expression of spinal PI3K-p110γ and p-AKT by CCI after posttreatment with EphB1-Fc. *P<0.05 or ** *P*<0.01 compared with Sham -Sal group, ^#^ *P*<0.05 compared with CCI-Sal group, n = 4 mice in each group.

We also observed the similar antinociceptive effect when EphB1-Fc was injected in neuropathic pain model. Consecutive intrathecal injection of EphB1-Fc on 1 h before CCI and days 1, day3 after CCI (0.5 µg for each injection) prevented the induction of thermal hyperalgesia and mechanical allodynia and this effect continued at least for 7 days after CCI (*P*<0.05 or *P*<0.01, EphB1-Fc-CCI group compared with Sal-CCI group; fig. 6A). On day 5 after CCI, a peak time point of pain behaviors, L4–L5 spinal cord of some mice were extracted for measurement of Fos protein, PI3K-p110γ and p-AKT expression. Injection of EphB1-Fc significantly inhibited the increased expression of spinal Fos protein (*P*<0.01 compared with Sal-CCI group; fig. 6B), PI3K-p110γ and p-AKT expression (PI3K-p110γ:Sal-CCI group compared with Sal-Sham group: *P*<0.01; EphB1-Fc-CCI group compared with Sal-CCI group: *P*<0.01;p-AKT: Sal-CCI group compared with Sal-Sham group: *P*<0.01; EphB1-Fc-CCI group compared with Sal-CCI group: *P*<0.05; fig. 6C) induced by CCI. Intrathecal injection of a single dose of EphB1-Fc (0.5 µg/5 µl) on the fifth day after CCI reversed the established thermal hyperalgesia and mechanical allodynia, and this effect could last up to 8 h (*P*<0.01,CCI-EphB1-Fc group compared with CCI-Sal group; fig. 6D). Spinal Fos protein (*P*<0.01 compared with CCI-Sal group; fig. 6E), PI3K-p110γ and p-AKT (PI3K-p110γ:CCI-Sal group compared with Sham-Sal group: *P*<0.05; CCI-EphB1-Fc group compared with CCI-Sal group: *P*<0.05;p-AKT: CCI-Sal group compared with Sham-Sal group: *P*<0.05; CCI-EphB1-Fc group compared with CCI-Sal group: *P*<0.05; fig. 6F) expression detected on 2 h after injection of EphB1-Fc also were markedly inhibited.

### PI3K activity is required for ERK activation induced by intrathecal injection of ephrinB1-Fc

**Figure 7:**
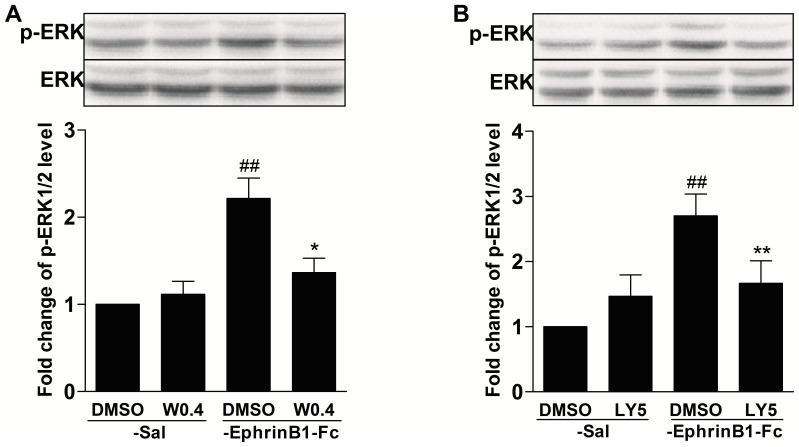
PI3K mediated spinal ERK activation induced by i.t. ephrinB1-Fc. PI3K inhibitor wortmannin (0.4 µg) or LY294002 (5 µg) or DMSO was intrathecally injected at 30 min before i.t. ephrinB1-Fc (0.5 µg). The expression of spinal p-ERK was assayed at 30 min after i.t. ephrinB1-Fc injection. Pre-treatment with both wortmannin (A) and LY294002 (B) inhibited spinal ERK activation induced by i.t. ephrinB1-Fc. The representative bands (top) and the quantitative data (bottom) for the expression of spinal p-ERK after i.t. ephrinB1-Fc (0.5 µg). The fold change for the density of each p-ERK normalized to ERK for each sample. The fold change of p-ERK level in DMSO-Sal group was set at 1 for quantifications. ^##^ *P*<0.01, compared with DMSO-Sal group, *P<0.05, **P<0.01 compared with DMSO-ephrinB1-Fc group, n = 6 mice in each group.

Previous and recent studies have shown that both peripheral and spinal ERK activation was involved in hyperalgesia induced by i.pl. or i.t. injection of ephrinB1-Fc [33], [34]. Pre- or post-treatment with MEK inhibitor prevented or reversed hyperalgesia induced by i.pl. or i.t. injection of ephrinB1-Fc.Moreover, ERK is a potential downstream target of PI3K and a recent study has shown that PI3K activity is required for ERK activation induced by i.pl. injection of ephrinB1-Fc in periphery [28]. Thus, we asked whether spinal PI3K pathway contributes to ERK activation induced by ephrinB1-Fc as the same as in periphery. To address this question, two kinds of PI3K inhibitor wortmannin (0.4 µg) or LY294002 (5 µg) at 30 min before ephrinB1-Fc injection was intrathecal administered. We measured activation of spnial ERK by western blot using a phosphopeptide specific antibody that recognizes phosphorylated ERK(p-ERK) at 30 min after ephrinB1-Fc injection. We found that both wortmannin and LY294002 prevented spinal ERK activation induced by ephrinB1-Fc(*P*<0.05 or P<0.01, compared with DMSO-ephrinB1-Fc group; fig. 7).

## Discussion

This study showed the following findings (1) spinal administration of ephrinB1-Fc produced a dose-dependent thermal hyperalgesia and mechanical allodynia, which was accompanied with the increased expression of spinal Fos protein and of spinal PI3K-p110γ expression as well as activation of AKT, a downstream target of PI3K.(2) inhibition of spinal PI3K prevented or reversed ephrinB1-Fc-induced hyperalgesia and spinal Fos protein expression;(3) blocking EphBs receptors alleviated formalin and CCI-induced pain behaviors and inhibited activation of spinal AKT; and (4) PI3K activity is required for ERK activation induced by i.t. injection of ephrinB1-Fc. These findings demonstrated PI3K/AKT signaling as a novel mechanism for ephrinBs/EphBs system involved in spinal nociceptive information modulation.

### ephrinBs/EphBs participate in central sensitization

Central sensitization, an activity-dependent functional plasticity in spinal cord neurons, is one of the main causes of behavior hyperalgesia under pathologic conditions and has been under intensive investigation [1], [2], [3]. Fos protein expression has been used as a maker for neuronal activation in the central nervous system and has been widely used as a tool in functional mapping of neuronal circuits in response to various defined stimuli. There is a positive correlation between the quantity of Fos protein expression and the degree of sensitization induced by nociceptive stimuli in spinal cord neurons. Long-term facilitation of c-fiber-evoked firing of wide dynamic range neurons in the spinal dorsal horn in response to conditioning stimulation of afferent fibers was accompanied with the frequency-dependent increase of c-fos-labeled cells in superficial, intermediate, and deep laminae of the dorsal horn on the stimulated side [35]. Inhibition of tetanically sciatic stimulation-induced LTP of spinal neurons was also accompanied with the suppressed tetanic stimulation-induced spinal Fos expression [36]. In this study, we further confirmed the previous electrophysiologic findings that activation of ephrinBs-EphBs receptor signaling is required for induction of c-fibers-evoked LTP of dorsal horn neurons and lowers stimuli threshold of the LTP [17] by means of Fos protein expression. Spinal application of ephrinB1-Fc, which can activate EphBs receptors, induced a dose-dependent increase in spinal Fos protein expression. Blocking of EphBs receptors with intrathecal injection of EphB1-Fc also suppressed the increased expression of spinal Fos protein expression in CCI and formalin pain models. Although we cannot identify which EphBs subgroup was activated dominantly by ephrinB1-Fc, our study demonstrates clearly that activation of ephrinBs/EphBs system is required for development of the pain-related,long-lasting alterations of excitability and synaptic plasticity of spinal neurons, which lead to increased sensitivity to noxious stimuli (hyperalgesia) or nonnoxious stimuli (allodynia).

EphBs receptors transduce signals bidirectionally by interacting with membrane-anchored ephrinBs ligands expressed on adjacent cells. Application of ephrinB1-Fc reagent can activate EphBs receptors (forward signaling) and also prevent activation of endogenous ephrinBs ligands (reverse signaling). EphB1-Fc can disturb the signaling by the interaction of EphBs and ephrinBs and activate endogenous ephrinBs ligands. In this study, we could not confirm whether reverse signaling produced by ephrinBs activation was involved in hyperalgesia induced by activation of spinal ephrinBs/EphBs signaling. However, injection of EphB1-Fc could not induce any pain behavior and not affect basal pain threshold. It is possible that reverse signaling has no contribution to this process, or EphB1-Fc induces only weak agonist activity. A recent finding in bone cancer model may support that EphB1 receptor forward signaling is critical to the development and maintenance of pain [37]. When EphB1 is inhibited, pain behaviors and NMDA receptors activation are inhibited even though the ephrinB2 continues to be active. When EphB1 receptor is activated, NMDARs are activated and pain is induced even though ephrinB2 is down regulated. But another recent study, by deleting ephrinB2 in Nav1.8^+^ nociceptive sensory neurons, indicates that presynaptic ephrinB2 expression may play a role in regulating inflammatory pain and some types of neuropathic pain through the regulation of synaptic plasticity in the DH [38].In conclusion, to date, we could confirm that the forword signaling produced by EphBs activation was involved in hyperalgesia induced by activation of ephrinBs/EphBs signaling. However, whether the reverse signaling produced by EphrinBs activation was involved in hyperalgesia induced by activation of ephrinBs/EphBs signaling still unclear.

### PI3K mediated the role of ephrinBs/EphBs in central sensitization

PI3K is widely expressed in spinal, particularly in the laminae I–II of the dorsal horn, the important sites for modulation of nociceptive information [24], [26], [39]. Ample and growing evidence showed that PI3K is involved in central sensitization induced by various stimuli [21], [22], [23], [24], [25], [26], [27]. Such as, Granulocyte-Colony Stimulating Factor (G-CSF) induces mechanical hyperalgesia via spinal activation of PI3K in mice [22]. Intradermal injection of capsaicin results in activation of PKB/Akt in the lumbar spinal cord [27]. Intrathecal wortmannin, a PI3K inhibitor, dose dependently attenuated carrageenan-induced thermal and tactile hyperalgesia and reversed an established thermal hyperalgesia when given as a posttreatment [40]. In Painful Inflammatory Conditions, Phosphatidylinositol 3-Kinase Is a Key Mediator of Central Sensitization, PI3K is required for the full expression of spinal neuronal wind-up, intrathecal administration of LY294002, a potent PI3K inhibitor, dose-dependently inhibited pain-related behavior and spinal application of LY294002 reduces the “wind-up” of deep dorsal WDR neurons and inhibits electrically evoked responses [24]. There also some evidence supports the notion that activation of central receptor tyrosine kinase (RTK) system and the related downstream signaling pathway contribute to the development of central sensitization [23], [25], [41]. For example, activation of TrkA, TrkB or G-CSF receptor tyrosine kinases by central administration of nerve growth factor (NGF), brain-derived neurotrophic factor (BDNF) or Granulocyte-Colony Stimulating Factor (G-CSF) produces hypersensitivity and pain in humans and reduces nociceptive threshold in animal models of pain. Blockage of NGF/TrkA, BDNF/TrkB system also attenuates excitability of neurons and prevents mechanical and thermal hyperalgesia [22], [42], [43], [44].Furthermore, PI3K activation mediates NFG, BDNF or C-GSF induced central sensitization and hyperalgesia [21], [22], [45].Moreover, several lines of evidence have shown that regulation of PI3K pathway is associated with EphBs receptors activation [28], [29], [30]. Some previous studies have indicated that ephrinBs acted as a sensitizer, which coupled with second-messenger systems through their EphBs receptors, to participate in central sensitization relevant to hyperalgesia [16], [17], [28], [33], [34]. In the present study, we found that ephrinB1-Fc-induced hyperalgesia was companied with the time- and dose-dependently increase of spinal PI3K-p110γ expression and activation of AKT. Increased phosphorylation of p-AKT was prevented by pre-treatment with PI3K inhibitors wortmannin and LY294002. Inhibition of spinal PI3K prevented and reversed pain behaviors and spinal Fos protein expression induced by i.t. injection of ephrinB1-Fc. blocking EphBs receptors alleviated formalin and CCI-induced pain behaviors and inhibited activation of spinal AKT. These results indicated that, like other RTK systems, pain behaviors induced by activation of spinal ephrinBs/EphBs signaling were mediated by activation of spinal PI3K.

### The crosstalk between PI3K and ERK signaling in ephrinBs/EphBs system

Some recent studies [28], [33], [34] and our results suggested that both peripheral and central ERK or PI3K activation are involved in pain behaviors induced by activation of peripheral and central ephrinBs/EphBs system. ephrinB1-Fc-induced hyperalgesia was companied with the time- and dose-dependently increase of p-ERK or PI3K expression. Inhibition of ERK or PI3K prevented and reversed pain behaviors and spinal Fos protein expression induced by i.pl. or i.t. injection of ephrinB1-Fc. pre-treatment with EphB1-Fc, which inhibited EphBs receptors, suppressed the pain behaviors and the increase of p-ERK or PI3K expression induced by inflammation and neuropathic pain. Furthermore, PI3K inhibitors blocked ERK activation induced by i.pl. or i.t. injection of ephrinB1-Fc. Our and other results support the crosstalk between PI3K and ERK signaling is involved in ephrinB1-Fc-induced pain model.

Two recent studies have showed that peripheral and Spinal NMDA receptor contributed to activation of ERK by ephrinB1-Fc [33], [34]. Previous results indicated that NMDA receptor-mediated [Ca^2+^]i increase can activate ERK pathways in a PI3K dependent manner [46], [47]. Therefore, we thought that exogenous ephrinBs-Fc or increased expression of ephrinBs by tissue or inflammation directly activates EphBs receptors in nociceptors or neuronal cells. Activated EphBs receptors can modulate the NMDA receptor function via a Src-dependent mechanism and then enhance NMDAR-mediated Ca^2+^. This [Ca^2+^]i elevation in turn directly facilitates ERK and AKT signaling via PI3K pathways. The activated ERK and AKT signaling further phosphorylated NMDA receptor [48], which increases membrane excitability and induces nociceptor sensitization or neuron exciting. On the other hand, the activated ERK and AKT signaling could modulate the expression of transcriptional factors, such as CREB and Fos, and promotes the transcription of downstream genes. The increased expression of pain-related genes may contribute to the maintenance of ephrinBs/EphBs signaling-induced hyperalgesia.

## Conclusion

This study demonstrates that activation of spinal ephrinBs/EphBs system induces hyperalgesia through a PI3K-mediated mechanism. These findings may have important implications for exploring the roles and mechanisms of ephrinBs/EphBs system underlying central nervous system diseases and for understanding the molecular basis for underlying physiologic and pathologic pain. In addition, it suggests that ephrinBs/EphBs pathway would be a new potential target for treatment of pathologic pain and other CNS diseases.

## Materials and Methods

### Animals

Adult, male Kunming mice (20–25 g) were employed in the present study. Mice were housed under a 12-h/12-h light–dark cycle regime, with free access to food and water. The animals were provided by Experimental Animal Center of Zhejiang University. All experimental protocols were approved by the Animal Care and Use Committee of Zhejiang University (Hangzhou, Zhejiang Province, China) and were in accordance with the Declaration of the National Institutes of Health Guide for Care and Use of Laboratory Animals (Publication No. 80-23, revised 1996).

### Drug application

Wortmannin, an irreversible PI3K inhibitor, and LY294002, a reversible competitive PI3K inhibitor were purchased from Sigma(St. Louis, MO). Both PI3K inhibitors were dissolved in 1% DMSO.ephrinB1-Fc and EphB1-Fc was purchased from R&D Systems Inc. (Minneapolis, MN) and dissolved in saline. The dosages of drugs are based on the results of preliminary experiments. The usage and dose for each drug were presented in the parts of results and figure legends. The method described by Hylden and Wilcox [49]was used for an intrathecal injection of drugs. In brief, a 28-gauge stainless steel needle attached to a 25-µl Hamilton microsyringe was inserted between the L5 and L6 vertebrae in conscious mice. A sudden slight flick of the tail indicated entry into the subarachnoid space. A volume of 5 µl of drug solution or physiologic saline was injected over a 30-s period into the subarachnoid space, and the injection cannula was left in place for a further 15 s. Motor function was evaluated by observation of placing or stepping reflexes and righting reflexes at 2 min before nociceptive test. Animals with signs of motor dysfunction were excluded from the experiments.

### Formalin Test

The procedure used was essentially the same as that reported by Hunskaar and Hole [50]. Approximately 30 min before testing, mice were individually placed in perspex observation chambers (10×20×15 cm) for adaptation. Then, the animals were taken out of the chamber, and 10 µl of 1% formalin in 0.9% saline was injected subcutaneously into the dorsal surface of the right hind paw with a 25-µl Hamilton syringe with a 28-gauge needle. Following injection, mice were immediately returned to the observation chamber. The amount of time spent licking and biting the injected paw and the number of flinching paw were measured from 0 to 5 min (the first phase) and from 10 to 40 min (the second phase) after formalin injection and was considered as indicative of nociception.

### Chronic Constrictive Injury Model

Chronic constrictive injury (CCI) model was performed following the method of Bennett and Xie [51]. In brief, mice were anesthetized with sodium pentobarbital (40 mg/kg, intraperitoneal injection). Left sciatic nerve was exposed at mid-thigh level through a small incision, and a unilateral constriction injury just proximal to the trifurcation was performed with three loose ligatures using a 5-0 silk thread (spaced at a 1-mm interval). In sham-operated animals, the nerve was exposed but not ligated. The incision was closed in layers,and the wound was treated with antibiotics.

### Measurement of Thermal Hyperalgesia

Thermal hyperalgesia was measured using the paw-withdrawal latency (PWL) according to the method described by Hargreaves et al. [52] In brief, mice were individually placed in clear plastic chambers (7×9×11 cm) and allowed to acclimatize to the environment for 1 h before testing. The radiant heat was directed to the plantar surface of each hind paw that was flushed against the glass or site of injection of solution through the glass plate. The nociceptive endpoints in the radiant heat test were the characteristic lifting or licking of the hind paw. The time to the endpoint was considered as the PWL. The radiant heat intensity was adjusted to obtain basal PWL of 12–15 s. An automatic 20-s cutoff was used to prevent tissue damage. Thermal stimuli were delivered three times to each hind paw at 5-min intervals.In CCI model, withdrawal latencies were measured in the same animal on both the ipsilateral (ligated) and the contralateral (unligated) paws. Results were expressed as the difference scores of PWL (s) = contralateral latency-ipsilateral latency.

### Measurement of Mechanical Allodynia

Mechanical allodynia was measured using the paw-withdrawal threshold (PWT) according to the method described by Cristiane F. Villarreal et al. [53] Mechanical hypernociception was evaluated in mice using the Dynamic Plantar Aesthesiometer (Ugo Basile) test. This apparatus has a pressure transducer coupled to a digital force detector that records the applied force in grams. Animals were placed in individual plastic boxes (20×25×15 cm) on a metal mesh floor and allowed to acclimate for 1 h. The test consists of evoking a hind-paw flexion reflex using a filament containing Universal Tip that touches the plantar surface and exerts an upward force (maximal 50 g) on the plantar surface of the mouse hind paw. The end point was defined by the withdrawal of the paw followed by clear flinching movements. With paw withdrawal, the recorded force was automatically displayed. The results are expressed by the intensity of hypernociception (in grams), Measurements were taken in triplicate and the average was taken as paw withdrawal threshold in grams. In CCI model, withdrawal thresholds were measured in the same animal on both the ipsilateral (ligated) and the contralateral (unligated) paw. Results were expressed as the difference scores of PWT (g) = contralateral threshold-ipsilateral threshold. The behavioral testing was performed by an investigator blinded to the treatment.

### Immunohistochemistry

Mice were anesthetized with sodium pentobarbital (60 mg/kg,intraperitoneal injection) and were subjected to sternotomy followed by intracardial perfusion with 20 ml saline and 100 ml 4% ice-cold paraformaldehyde in 0.1 mol/l phosphate buffer(PB). The spinal cord of L4–L5 was removed, postfixed in 4% paraformaldehyde overnight, and subsequently allowed to equilibrate in 30% sucrose in PB overnight at 4°C. Thirty micrometer transverse series sections were cut on a cryostat and stored in phosphate buffer. After washing in phosphate buffer saline, the tissue sections were incubated in phosphate buffer saline containing 5% normal goat serum and 0.3% Triton X-100 at room temperature for 30 min. For the Fos protein assay, the sections were incubated in primary polyclonal rabbit-anti-Fos antibody (1∶1,000; Santa Cruz Biotechnology, Santa Cruz, CA) at 4°C for 48 h. The sections were then incubated in biotinylated goat anti-rabbit (1∶200) at 37°C for 1 h and in avidin-biotin-peroxidase complex (1∶100; Vector Labs, Burlingame, CA) at 37°C for 2 h. Finally, the sections were treated with 0.05% diaminobenzidine for 5–10 min. Sections were rinsed in phosphate buffer saline to stop the reaction, mounted on gelatin-coated slides, air-dried, dehydrated with 70–100% alcohol, cleared with xylene, and cover slipped for microscopic examination.For analysis of the change of Fos protein expression, we examined five L4–5 spinal cord sections per animal, selecting the sections with the greatest number of positive neurons.For each animal, we counted the total number of positive neurons in the bilateral spinal cord I–V and X lamina, regardless of the intensity of the staining.

### Western Blot Analysis

The spinal cords of mice were quickly extracted and stored in liquid nitrogen. Tissue samples were homogenized in lysis buffer(pH 7.4) containing (in millimoles): Tris, 20.0 mM; sucrose,250.0 mM; Na3VO4, 0.03 mM,MgCl2, 2.0 mM, EDTA, 2.0 mM, EGTA, 2.0 mM; phenylmethylsulfonyl fluoride, 2.0 mM; dithiothreitol, 1.0 mM; protease inhibitor cocktail,0.02% (v/v). The homogenates were centrifuged at 5,000 g for 30 min at 4°C. The supernatant was collected,and protein concentration was measured according to the Bradford [54] method, using bovine serum albumin as standard. The protein samples were stored at −80°C.Protein samples were dissolved in 4×sample buffer (pH 6.8) containing: Tris–HCl, 250.0 mM; sucrose, 200.0 mM; dithiothreitol, 300.0 mM; Coomassie brilliant blue-G, 0.01%; and sodium dodecyl sulfate, 8% and denatured at 95°C for 5 min, then the equivalent amounts of proteins (30 µg) were separated by using 10% sodium dodecyl sulfate-polyacrylamide gel electrophoresis and transferred onto 0.45 µm Polyviny lidene fluoride(PVDF) membrane.In addition, the gels stained with Coomassie blue were used to confirm the equal amounts of protein loaded on each lane. The membranes were incubated overnight at 4°C with the following primary antibodies: primary polyclonal rabbit anti-PI3K-p110γ antibody(1∶100), primary polyclonal rabbit anti-p-AKT or anti-AKT antibody (1∶1000), primary polyclonal rabbit anti-p-ERK1/2 or anti-ERK1/2 antibody (1∶1000), or primary polyclonal rabbit-anti-GAPDH antibody (1∶10,000). The specificity for these antibodies was confirmed by loss of bands in the absence of primary antibodies.The membranes were extensively washed with Tris-buffered saline Tween20 and incubated for 1 hour with the secondary antibody conjugated with Horseradish peroxidase (HRP) (1∶10,000) at room temperature. The immune complexes were detected by using ECL western detection reagents. Western blot densitometry analysis of signal intensity was performed using Quantity One 4.6.2 (Bio-Rad; Hercules, CA) and phosphorylation levels of AKT from densitometry were normalized to AKT. The blot density from control groups was set as 100%.

### Statistical Analysis

Data are expressed as mean ± SEM. Statistical analysis between two samples was performed by Student *t* test. Statistical comparison of more than two groups was performed using one-way ANOVA followed by a Tukey *post hoc* test. The significance of any differences in thermal latency and mechanical threshold in behavior test was assessed using two-way ANOVA. “Time” was treated as “within subjects” factor and “treatment” was treated as a “between subjects” factor. The area under the pain threshold change versus time curve was calculated by GraphPAD Prism5 (Graph Pad Software, Inc., San Diego, CA) in some behavioral test. Statistical analyses of data were generated by GraphPAD Prism 5. All *P* values given are based on two-tailed tests. A value of *P* less than 0.05 was considered as statistically significant.
